# Effects of dietary astaxanthin supplementation on growth performance, antioxidant status, immune response, and intestinal health of rainbow trout (*Oncorhynchus mykiss*)

**DOI:** 10.1016/j.aninu.2024.03.010

**Published:** 2024-03-30

**Authors:** Xiaoxue Meng, Fumei Yang, Lulu Zhu, Lingli Zhan, Toru Numasawa, Junming Deng

**Affiliations:** a College of Fisheries, Guangdong Ocean University, Zhanjiang 524088, China; b Kunming Biogenic Co., Ltd., Kunming 650220, China

**Keywords:** Astaxanthin, Growth, Antioxidant, Immune, Intestinal health

## Abstract

A feeding trial was conducted to assess the impacts of dietary astaxanthin from wall-broken *Haematococcus pluvialis* (WBHPA) on the growth performance, antioxidant status, immune response, and intestinal health of rainbow trout (*Oncorhynchus mykiss*). Six experimental diets were formulated with various concentrations of WBHPA, ranging from 0 to 8.4 g/kg (containing 0 to 125 mg/kg astaxanthin). Each diet was fed to triplicate groups of rainbow trout (mean initial weight of 561 g) twice daily for 9 consecutive weeks. The survival rate and feed intake of fish exhibited no significant differences among the dietary groups (*P* > 0.05). Similarly, dietary inclusion of 25 to 100 mg/kg astaxanthin did not significantly affect the weight gain and daily growth coefficient (*P* > 0.05), but excessive inclusion of astaxanthin (125 mg/kg) slightly depressed these parameters (*P* < 0.05). Dietary inclusion of 25 to 50 mg/kg astaxanthin increased the activities of intestinal digestion and absorption enzymes (lipase, creatine kinase, and alkaline phosphatase), while the inclusion of 25 to 75 mg/kg astaxanthin improved the immune response of fish. Furthermore, regardless of inclusion level (25 to 125 mg/kg), dietary astaxanthin supplementation strengthened the intestinal mucosal barrier function and improved antioxidant activity, thereby promoting intestinal development. Conclusively, 25 to 75 mg/kg astaxanthin from WBHPA was recommended to be included in diets for rainbow trout.

## 1 Introduction

Astaxanthin, a type of carotenoid, can promote fish pigmentation (Pan and Chien, 2009; Yi et al., 2014) and exerts a crucial role in enhancing the antioxidant capacity, stress resistance, and immune response of fish (Zhang et al., 2013). Fish cannot synthesize carotenoids de novo (Goodwin, 1984) and therefore rely on dietary intake (Bahremand et al., 2012). There are two primary sources of astaxanthin: natural and synthetic. It is important to note that these two sources of astaxanthin have different effects owing to their disparity of structure. Natural astaxanthin is mainly found in the esterified form, while synthetic astaxanthin, known for its significant instability, exists in the free form (Ambati et al., 2014). Although synthetic astaxanthin offers cost advantages, it is worth acknowledging that astaxanthin originating from green microalga *Haematococcus pluvialis* exhibits superior antioxidant capacity due to its fully levorotatory structure (Liu et al., 2016; Zhao et al., 2023).

Due to its high accumulation rate of astaxanthin, *H. pluvialis* is considered the primary source with the highest natural content of this pigment. The extracted astaxanthin ester ratio from *H. pluvialis* (70% monoester, 25% diester, and 5% monomer) closely resembles that in aquatic animals, which enhances its bioavailability potential. Previous research has shown that the inclusion of *H. pluvialis* in the diet for large yellow croaker (*Pseudosciaena crocea*) at levels ranging from 2800 to 5600 mg/kg can remarkably strengthen the antioxidant capacity, immune response, and general health (Li et al., 2014). It has also shown superior growth-promoting efficacy as compared to synthetic astaxanthin. Similar effects have been observed in olive flounder (*Paralichthys olivaceus*) when supplemented with 100 mg/kg of *H. pluvialis* in their diet, with comparable results to synthetic astaxanthin (Pham et al., 2014). However, several studies have reported that the efficacy of *H. pluvialis* supplementation in the diet is lower than that of synthetic astaxanthin (Pan and Chien, 2009). This difference is likely attributed to the presence of cell walls in *H. pluvialis*, which restricts nutrient uptake and utilization. Therefore, addressing the challenge associated with cell wall composition is crucial for improving the utilization efficiency of *H. pluvialis*.

Rainbow trout (*Oncorhynchus mykiss*), a significant carnivorous cold-water fish species, has been extensively cultivated with a global production of 959,600 tonnes achieved in 2020 (FAO, 2020). With the sustained development of aquaculture, the expansion of intensive aquaculture is inevitable and will exert greater pressure on fish. In this context, it is crucial to develop additives with prebiotic effects that can effectively balance nutrition utilization, promote growth performance, and maintain fish health. This research systematically and comparatively investigates the impact of astaxanthin derived from wall-broken *H. pluvialis* (WBHPA) on the growth, antioxidant status, immune response, and intestinal health of rainbow trout. We sought to provide insights into efficient utilization strategies for *H. pluvialis* and establish a scientific foundation for incorporating dietary astaxanthin in rainbow trout farming practices.

## 2 Materials and methods

### 2.1 Animal ethics statement

The experimental protocols were approved by the Institutional Animal Care and Use Committee of Guangdong Ocean University (approval ID: GDOU-IACUC−2020-A0926). All experimental procedures were conducted in strict accordance with the guidelines provided by the committee.

### 2.2 Experimental diets

A basal diet for rainbow trout (crude protein 40%, crude lipid 18%) was formulated with fish meal, soybean meal, and rapeseed meal as the primary protein sources, and soybean oil, fish oil, and soybean lecithin as the lipid sources (Table 1). To create six experimental diets with varying astaxanthin concentrations (0, 25, 50, 75, 100, and 125 mg/kg), graded levels of WBHPA (purity 1.5%, provided by Kunming Biogenic Co., Ltd., Kunming, China) were added to the basal diet. Correspondingly, these diets were designated as control, WBHPA-25, WBHPA-50, WBHPA-75, WBHPA-100, and WBHPA-125, respectively.

**Table 1:** Ingredients and proximate composition of experimental diets.

Item	Control	WBHPA-25	WBHPA-50	WBHPA-75	WBHPA-100	WBHPA-125
Ingredients, % DM						
Fish meal^1^	28.00	28.00	28.00	28.00	28.00	28.00
Soybean meal^1^	28.00	28.00	28.00	28.00	28.00	28.00
Rapeseed meal^1^	12.00	12.00	12.00	12.00	12.00	12.00
Wheat flour	2.18	2.01	1.85	1.68	1.51	1.34
α-Starch	12.00	12.00	12.00	12.00	12.00	12.00
Soybean oil	8.00	8.00	8.00	8.00	8.00	8.00
Fish oil	6.00	6.00	6.00	6.00	6.00	6.00
Soybean lecithin	0.50	0.50	0.50	0.50	0.50	0.50
WBHPA^2^	0.00	0.17	0.33	0.50	0.67	0.84
Vitamin C	0.02	0.02	0.02	0.02	0.02	0.02
Ca(H_2_PO_4_)_2_	1.00	1.00	1.00	1.00	1.00	1.00
Choline chloride (50%)	0.30	0.30	0.30	0.30	0.30	0.30
Vitamin mixture^3^	1.00	1.00	1.00	1.00	1.00	1.00
Mineral mixture^4^	1.00	1.00	1.00	1.00	1.00	1.00
Proximate composition						
DM, %	90.32	91.26	90.43	91.31	91.19	90.96
Crude protein, % DM	39.86	39.74	39.97	39.92	40.02	39.89
Crude lipid, % DM	17.97	17.99	18.09	18.26	18.30	18.26
Ash, % DM	10.07	9.87	9.84	9.71	10.05	9.79
Gross energy, MJ/kg DM	21.92	21.99	22.06	21.83	22.01	21.91
Astaxanthin, mg/kg DM	ND	24.56	48.60	72.15	95.19	117.72

DM = dry matter; WBHPA = wall-broken *Haematococcus pluvialis*; ND = not detected.

1 Supplied by Kunming Tianyuan Feed Co., Ltd. (Yunnan, China); fish meal, 72.31% crude protein, 10.00% crude lipid; soybean meal, 52.98% crude protein, 0.85% crude lipid; rapeseed meal, 42.93% crude protein, 1.51% crude lipid.

2 Supplied by Kunming Biogenic Co., Ltd. (Yunnan, China); the purity of astaxanthin from WBHPA was 1.5%. Additionally, WBHPA also included 40% cellulose, 25% dextrin, 15% protein, 15% lipid, and 3.5% phospholipid.

3 Vitamin premix (g/kg mixture): retinyl acetate (2,800,000 IU/g), 2; cholecalciferol, 0.03; DL-α-tocopheryl acetate, 30; menadione, 3; thiamine hydrochloride, 8; riboflavin, 11; pyridoxine hydrochloride, 8; vitamin B_12_, 0.02; ascorbic acid, 50; folic acid, 1; biotin, 0.1; niacin, 30; calcium D-pantothenate, 32; inositol, 25.

4 Mineral premix (g/kg mixture): MgSO_4_·7H_2_O, 180; KI, 1; FeSO_4_·H_2_O, 260; ZnSO_4_·H_2_O, 180; CuSO_4_·5H_2_O, 25; Na_2_Se_2_O_3_, 0.01; MnSO_4_·H_2_O, 180; CoCl_2_·6H_2_O, 0.75.

The dry ingredients were finely powdered through a 60-mesh sieve and thoroughly mixed with fish oil, soybean oil, and soybean lecithin. The mixture was then combined with approximately 30% distilled water. Next, the mixture was extruded using a pellet feed maker (KS-180, Jiangsu Jinggu Rice Mill Co., Ltd., Jiangsu, China) and filtered through a 3-mm die. Afterward, the pellets were dried at a constant temperature of 40 °C for 12 h and preserved at −20 °C before the feeding trial.

### 2.3 Experimental fish, feeding management, and sampling

Rainbow trout adopted in this experiment were hatched from the same batch and had an initial body weight of 561.49 ± 2.17 g. Prior to the feeding trial, the fish were given a commercial diet for 1 week to acclimate to the experimental conditions. At the start of the feeding trial, 540 fish were fasted for 24 h and then randomly distributed into 18 cement tanks. Each dietary group had three tanks, with 30 fish housed in each tank (2 m × 2 m × 1.5 m). The fish were hand-fed twice daily (at 07:00 and 17:00) until they appeared satiated, for 9 weeks. The water in the tanks was aerated, treated with dichlorination, and then filtered through a circulating water system that included mechanical and biological filtration media, as well as a UV lamp disinfection device. The water flow rate was maintained at 10 L/min. The tanks were continuously aerated and subjected to a natural photoperiod, with water temperatures ranging from 14 to 18 °C throughout the experiment. Additionally, the dissolved oxygen levels ranged from 7.8 to 9.2 mg/L, pH levels ranged from 6.7 to 7.7, total ammonia nitrogen levels ranged from 0.04 to 0.07 mg/L, and nitrite levels ranged from 0.02 to 0.04 mg/L.

After the feeding trial, all fish were fasted for 24 h. The fish in each tank were then anesthetized using eugenol (1:12,000 dilution; Shanghai Reagent Corporation, Shanghai, China). The fish in each tank were collected with a net and dried with a towel, then bulk-weighed and recorded the number to calculate the survival rate, weight gain (WG), daily growth coefficient (DGC), feed intake (FI), feed conversion ratio (FCR), and protein efficiency ratio (PER). Blood samples were collected using a 1-mL syringe and allowed to clot at 4 °C for 8 h. The supernatants were harvested after centrifugation (4000 × *g*, 10 min, 4 °C). Additionally, 3 fish were randomly selected from each tank, and their weight and body length were measured. After dissection, the visceral organs, intestines, and liver of fish were weighed individually. The length of the intestine was measured using a ruler. Furthermore, liver and intestinal samples were collected from 3 fish randomly chosen from each tank and then, immediately transferred to RNA-free enzyme tubes. All samples were rapidly frozen with liquid nitrogen and preserved at −76 °C until further analysis.

### 2.4 Analysis of proximate composition

The proximate composition analysis of experimental diets conformed to the standard methods of the Association of Official Analytical Chemists (AOAC, 2000). The moisture assay was achieved in an oven at 105 °C until a constant weight. Protein content was determined by measuring nitrogen (N × 6.25) using the Kjeldahl method. Lipid analysis was carried out using the Soxhlet method. Ash content was measured through incineration at 550 °C. Gross energy was determined using an oxygen bomb calorimeter (ZDHW-6, Hebi Huatai Electronics Co., Ltd., Henan, China). The astaxanthin concentration in the diets was determined using high-performance liquid chromatography (Agilent 1100 series, Agilent Technologies, USA) according to China National Standard GB/T 31520-2015.

### 2.5 Enzyme activities in plasma, liver, and intestine

The plasma samples were directly employed while tissue samples (liver and intestine) were prepared into a crude enzyme solution for the determination of the relevant biochemical indicators described in the previous report (Deng et al., 2021). The diamine oxidase (DAO) activity and the contents of endothelin-1 (ET-1), reactive oxygen species (ROS), interferon-gamma (IFN-γ), interleukin-1 beta (IL-1β), tumor necrosis factor-alpha (TNF-α), IL-6, IL-8, IL-10, and transforming growth factor-beta 1 (TGF-β) were measured by corresponding enzyme-linked immunosorbent assay (ELISA) commercial kits following the instructions (R&D Systems, Inc., Minneapolis, USA), as discussed by Deng et al. (2020, 2023).

The Folin-phenol method was employed to determine the intestinal trypsin activity. The activities or contents of lipase, amylase, creatine kinase, Na^+^–K^+^-ATPase, alkaline phosphatase (AKP), superoxide dismutase (SOD), catalase (CAT), glutathione peroxidase (GSH-Px), total antioxidant capacity (TAC), glutathione (GSH), malondialdehyde (MDA), immunoglobulin M (IgM), lysozyme (LZM), and total protein were measured by means of corresponding commercial kits as per the instructions (No. A054-2-1, No. C016-1-1, No. A032-1-1, No. A070-2-2, No. A059-2-2, No. A001-3-2, No. A007-2-1, No. A005-1-2, No. A015-2-1, No. A006-2-1, No. A003-1-2, No. H109-1-2, No. A050-1-1, and No. A045-2-2, respectively; Nanjing Jiancheng Bioengineering Institute, Nanjing, China).

### 2.6 Histological observation

The hindgut intestines were immersed in 10% neutral formalin for 24 h and subsequently underwent dehydration, embedding, sectioning, and spreading following the standard procedures described by Liu et al. (2022). The intestinal sections were stained with hematoxylin and eosin (H&E) and visualized under an optical microscope (Leica Application Suite).

### 2.7 RNA extraction and real-time quantitative PCR (qRT-PCR) analysis

After extracting total RNA from the liver and intestine (TaKaRa Biotechnology Co., Ltd., Japan), the RNA was reverse transcribed to cDNA with the application of the PrimeScript RT reagent Kit (TaKaRa Biotechnology Co., Ltd., Japan). Specific primers for target genes and the reference gene (18S rRNA) were designed based on the GenBank database (Table 2). The PCR efficiency of all primers was measured following the protocol described by Meng et al. (2022). Subsequently, the designed primers were synthesized by Tsingke Biotech Co., Ltd. (Beijing, China). The reaction system (25 μL in total) consisted of 2 μL of cDNA template, 12.5 μL of SYBR Premix Ex Taq II (Tli RNaseH Plus, TaKaRa Biotechnology Co., Ltd., Japan), 1 μL of forward primer (10 μmol/L), 1 μL of reverse primer (10 μmol/L), and 8.5 μL of sterilized water. Each sample was analyzed in triplicate. The mRNA expression levels were quantified using qRT-PCR based on the 2^−ΔΔCt^ method (Livak and Schmittgen, 2001).

**Table 2:** Primers used for quantitative real-time PCR analysis.

Name	Primer sequence (5′ to 3′)	Length, bp	Tm, °C	Accession number	PCR efficiency, %
*pIgR*	F: CAGAAGAAAGCCCTCAGTCTGT	22	61.5	XM_036966765.1	104.3
	R: GTCTTGTCCTGTGGGTTTTGTT	22			
*IL-10*	F: TGGACAGCATCCTGAAGTTCTA	22	60.5	NM_001245099.1	95.94
	R: AGTAGTTCCTGCATTGGACGAT	22			
*TGF-β*	F: GTGAGAACACCAGTAAGCACCA	22	62.0	XM_021591332.2	96.84
	R: CTTGGAGACAAAATGGGACTTC	22			
*IL-1β*	F: TATGAAGAACTCCCACACTATCCA	24	60.3	NM_001124347.2	98.36
	R: TTTGGTTGTCATGTAGAAGAGGAA	24			
*IL-6*	F: ACTCCCCTCTGTCACACACC	20	63.1	NM_001124657.1	91.71
	R: GGAAGTCTTTGCCCCTCTTT	20			
*IL-8*	F: CCAAGATGAGCATCAGAATGTC	22	58.4	XM_021614893.2	94.43
	R: CTCAGAGTGGCAATGATCTCAG	22			
*IL-12β*	F: GCCAACATGAGACCATTGTG	20	58.8	XM_036937824.1	93.05
	R: CTCCCATCATCTCCACCACT	20			
*IFN-γ*	F: ATGCTGCTCAGTTCACATCAAT	22	60.2	NM_001160503.1	97.24
	R: TCAGATACACGTCCAGAACCAC	22			
*TNF-α*	F: CCTTGAAAATAGCCTTGTGGAC	22	58.9	NM_001124357.1	95.62
	R: TTGCACCAATGAATATCTCCAG	22			
18S rRNA	F: TAACGAACGAGACTCCGGCA	20	62.8	NC_048575.1	94.15
	R: GTTCATCGGGTTACCCACGC	20			

*pIgR* = polymeric immunoglobulin receptor; *IL* = interleukin; *TGF-β* = transforming growth factor-beta 1; *IFN-γ* = interferon-gamma; *TNF-α* = tumor necrosis factor-alpha.

### 2.8 Statistical analysis

The normality and homogeneity of the variance were tested. Then the one-way analysis of variance (ANOVA) in SPSS 17.0 for Windows (percentage data were firstly arcsine transformed) was conducted in this research. Tukey's multiple comparisons were employed to identify significant differences between groups. A significance level of *P* < 0.05 was considered. The results are presented as means ± standard error across all groups.

## 3 Results

### 3.1 Growth performance

**Fig. 1:**
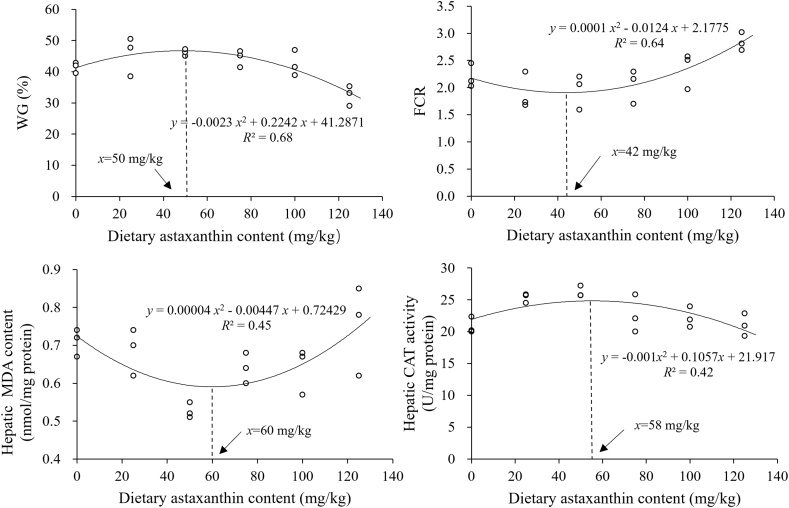
Regression analysis between dietary astaxanthin content (mg/kg) and weight gain (WG, %), feed conversion ratio (FCR) as well as hepatic malondialdehyde (MDA) content (mmol/mg protein) and catalase (CAT) activity (U/mg protein) in rainbow trout fed diets with various levels of astaxanthin from wall-broken *Haematococcus pluvialis*.

After 9 weeks of feeding, all fish appeared to be healthy and active. No significant difference was observed in the FI among the dietary groups (*P* > 0.05; Table 3). The final body weight, WG (Fig. 1), and DGC initially increased and then decreased with the increase of dietary WBHPA concentration, with the lowest values found in the WBHPA-125 group. Similarly, PER first increased and then decreased with the increase of dietary WBHPA concentration, with the highest value observed in the WBHPA-25 group. However, the FCR (Fig. 1) exhibited the opposite changes. Furthermore, with the increase of dietary WBHPA concentration, the hepatosomatic index and viscerosomatic index were firstly elevated and then reduced, and the highest values were detected in the WBHPA-50 group.

**Table 3:** Growth performance of rainbow trout fed diets with various levels of astaxanthin from wall-broken *Haematococcus pluvialis* (WBHPA).

Item	Control	WBHPA-25	WBHPA-50	WBHPA-75	WBHPA-100	WBHPA-125
Initial body weight, g	555.70 ± 23.74	569.80 ± 10.00	561.30 ± 13.48	556.34 ± 12.24	562.26 ± 21.94	565.90 ± 15.25
Final body weight, g	786.03 ± 9.59^ab^	829.46 ± 13.47^b^	820.68 ± 3.15^b^	803.31 ± 10.49^b^	800.93 ± 11.81^b^	749.88 ± 14.57^a^
Weight gain, %	41.45 ± 1.73^b^	45.57 ± 2.36^b^	46.21 ± 0.56^b^	44.39 ± 1.89^b^	42.45 ± 2.10^b^	32.51 ± 2.57^a^
DGC, %/d	1.60 ± 0.06^b^	1.75 ± 0.08^b^	1.77 ± 0.02^b^	1.70 ± 0.06^b^	1.64 ± 0.07^b^	1.29 ± 0.09^a^
FI, g/kg MBW per day	10.51 ± 0.70	10.47 ± 0.67	10.39 ± 0.34	10.50 ± 0.27	10.83 ± 0.43	10.92 ± 0.49
Feed conversion ratio	2.20 ± 0.13^ab^	1.90 ± 0.20^a^	1.95 ± 0.18^a^	2.05 ± 0.18^ab^	2.35 ± 0.19^ab^	2.84 ± 0.10^b^
Protein efficiency ratio	1.15 ± 0.04^abc^	1.34 ± 0.06^c^	1.29 ± 0.05^bc^	1.22 ± 0.06^bc^	1.05 ± 0.08^ab^	0.90 ± 0.07^a^
Hepatosomatic index, %	0.69 ± 0.04^a^	1.09 ± 0.10^b^	1.13 ± 0.08^b^	1.08 ± 0.05^b^	1.09 ± 0.09^b^	0.95 ± 0.03^ab^
Viscerosomatic index, %	7.82 ± 0.25^a^	9.52 ± 0.67^ab^	10.56 ± 0.56^b^	8.85 ± 0.17^ab^	7.22 ± 0.51^a^	7.58 ± 0.50^a^

DGC = daily growth coefficient; FI = feed intake; MBW = mean metabolic body weight.

Values are presented as means ± SE (*n* = 3). ^a,b,c^ Means in the same row with different superscripts are significantly different (*P* < 0.05).

Weight gain (%) = 100 × (final body weight – initial body weight)/initial body weight.

DGC (%/d) = 100 × (final body weight^1/3^ – initial body weight^1/3^)/day.

MBW = [(initial body weight/1000)^0.75^ + (final body weight/1000)^0.75^]/2.

FI (g/kg MBW per day) = feed consume/(MBW × day).

Feed conversion ratio = total feed intake/(final body weight – initial body weight).

Protein efficiency ratio = (final body weight – initial body weight)/total protein intake.

Hepatosomatic index (%) = 100 × hepatic weight/body weight.

Viscerosomatic index (%) = 100 × visceral weight/body weight.

### 3.2 Intestinal development

**Fig. 2:**
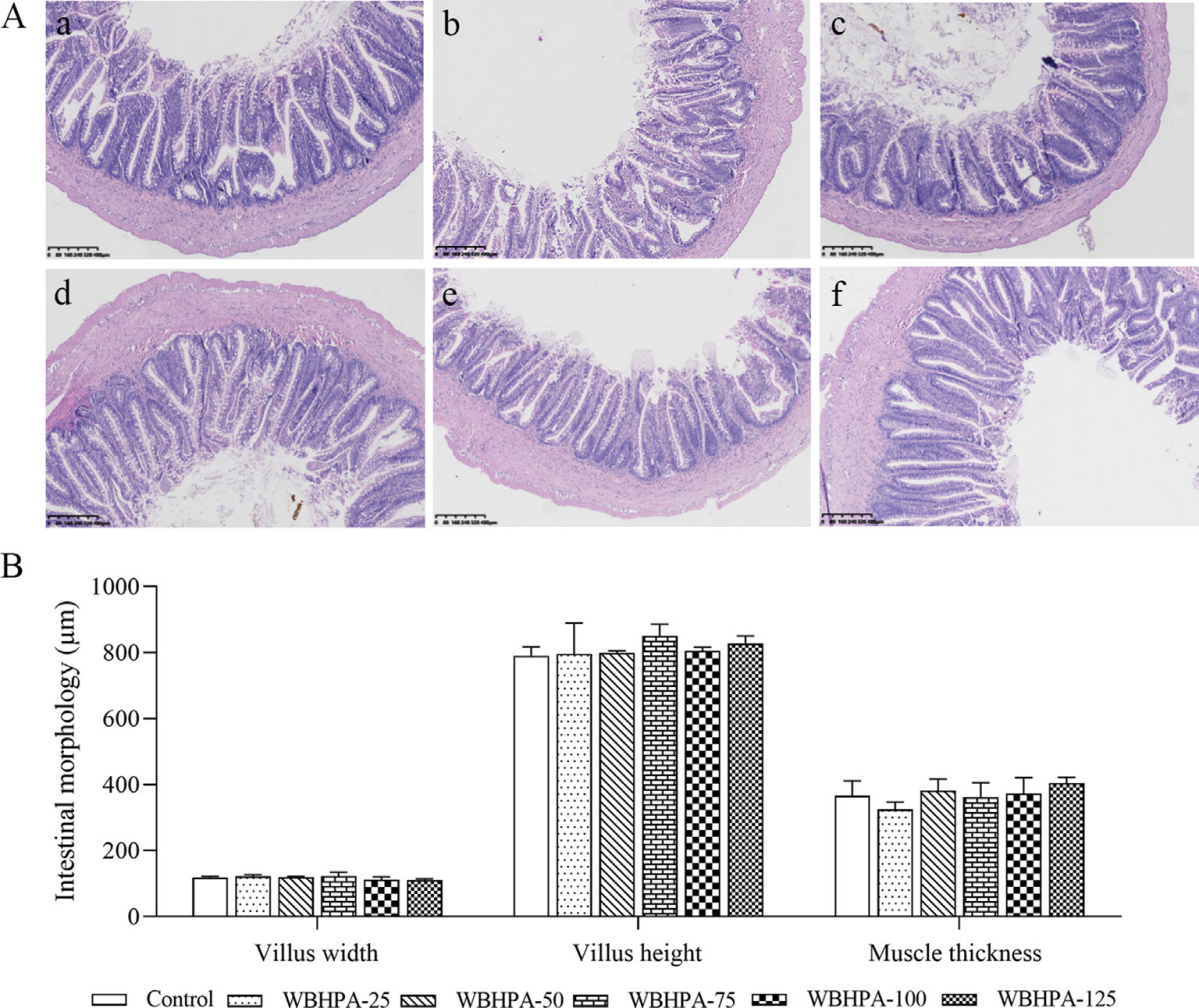
Morphological structure of hindgut in rainbow trout fed diets with various levels of astaxanthin from wall-broken *Haematococcus pluvialis* (WBHPA). (A) Histological sections of rainbow trout fed with the (a) control, (b) WBHPA-25, (c) WBHPA-50, (d) WBHPA-75, (e) WBHPA-100, and (f) WBHPA-125 diet, respectively, with H&E staining. Scale bar, 400 μm. Magnification, 80×. (B) Intestinal morphology parameters (means ± SE).

The relative intestinal length of rainbow trout exhibited an insignificant difference among the dietary groups (*P* > 0.05; Table 4). With the increase of dietary WBHPA concentration, the relative intestinal weight and the intestinal density first increased and then decreased, and the highest values were observed in the WBHPA-25 group. In addition, the morphological structure of hindgut in rainbow trout was not notably affected by the dietary content of astaxanthin (*P* > 0.05, Fig. 2).

**Table 4:** Intestinal development of rainbow trout fed diets with various levels of astaxanthin from wall-broken *Haematococcus pluvialis* (WBHPA).

Item	Control	WBHPA-25	WBHPA-50	WBHPA-75	WBHPA-100	WBHPA-125
Intestinal relative length, %	1.40 ± 0.04	1.51 ± 0.01	1.52 ± 0.04	1.52 ± 0.10	1.53 ± 0.05	1.54 ± 0.12
Intestinal relative weight, %	0.54 ± 0.01^bc^	0.65 ± 0.06^c^	0.45 ± 0.01^ab^	0.38 ± 0.04^ab^	0.34 ± 0.01^a^	0.42 ± 0.04^ab^
Intestinal density, g/cm	39.11 ± 2.44^bc^	42.97 ± 3.71^c^	29.26 ± 1.11^ab^	24.40 ± 1.03^a^	21.87 ± 1.10^a^	27.16 ± 2.60^a^

Values are presented as means ± SE (*n* = 3). ^a,b,c^ Means in the same row with different superscripts are significantly different (*P* < 0.05).

The intestinal relative length (%) = 100 × intestinal length/body length.

The intestinal relative weight (%) = 100 × intestinal weight/body weight.

The intestinal density (g/cm) = intestinal weight/intestinal length.

### 3.3 Intestinal digestive and absorption enzyme activities

No noticeable differences were observed regarding the intestinal trypsin, amylase, and Na^+^–K^+^-ATPase activities of rainbow trout among the dietary groups (*P* > 0.05; Table 5). Regardless of the supplemented concentration (25 to 125 mg/kg), dietary astaxanthin supplementation increased intestine lipase activity, while that was markedly higher in the WBHPA-25 group (*P* < 0.05). With the increase of dietary WBHPA concentration, the activities of intestinal creatine kinase and AKP first increased and then decreased, with the highest values in the WBHPA-50 and WBHPA-25 groups, respectively.

**Table 5:** Intestinal digestion and absorption enzymes activities of rainbow trout fed diets with various levels of astaxanthin from wall-broken *Haematococcus pluvialis* (WBHPA).

Item	Control	WBHPA-25	WBHPA-50	WBHPA-75	WBHPA-100	WBHPA-125
Trypsin, U/mg protein	3.22 ± 0.74	2.85 ± 0.53	2.53 ± 0.15	2.51 ± 0.41	3.03 ± 0.37	3.10 ± 0.95
Lipase, U/g protein	5.86 ± 0.70^a^	20.53 ± 1.18^b^	8.20 ± 1.02^a^	7.83 ± 0.51^a^	7.98 ± 0.42^a^	8.59 ± 0.43^a^
Amylase, U/mg protein	0.29 ± 0.08	0.33 ± 0.03	0.41 ± 0.02	0.30 ± 0.05	0.41 ± 0.05	0.38 ± 0.04
Creatine kinase, U/g protein	51.51 ± 0.07^ab^	70.98 ± 9.16^ab^	77.52 ± 2.65^b^	55.06 ± 8.02^ab^	44.97 ± 3.75^a^	49.91 ± 7.62^ab^
Na^+^–K^+^-ATPase, U/mg protein	2.65 ± 0.12	2.65 ± 0.13	2.88 ± 0.41	3.00 ± 0.13	2.64 ± 0.21	2.55 ± 0.05
Alkaline phosphatase, U/g protein	67.65 ± 4.97^ab^	85.19 ± 0.66^b^	74.31 ± 8.67^ab^	51.05 ± 3.96^a^	67.94 ± 5.86^ab^	62.49 ± 3.15^ab^

Values are presented as means ± SE (*n* = 3). ^a,b^ Means in the same row with different superscripts are significantly different (*P* < 0.05).

### 3.4 The intestinal mucosal barrier

No significant differences were detected in plasma and intestinal DAO activities as well as the intestinal ET-1 content in rainbow trout among dietary groups (*P* > 0.05; Table 6). The plasma ET-1 content initially exhibited reductions and then elevations with the increase of dietary WBHPA concentration. The lowest plasma ET-1 value was found in the WBHPA-100 group, which was noticeably lower than the control group (*P* < 0.05). Regardless of the supplemented concentration (25 to 125 mg/kg), dietary astaxanthin supplementation remarkably up-regulated the intestinal resistance value (*P* < 0.05).

**Table 6:** Intestinal mucosal barrier function of rainbow trout fed diets with various levels of astaxanthin from wall-broken *Haematococcus pluvialis* (WBHPA).

Item	Control	WBHPA-25	WBHPA-50	WBHPA-75	WBHPA-100	WBHPA-125
Plasma						
Endothelin-1, μg/mL	105.38 ± 2.79^c^	97.92 ± 2.92^bc^	93.89 ± 1.48^ab^	89.73 ± 1.80^ab^	86.29 ± 1.04^a^	94.60 ± 3.23^abc^
Diamine oxidase, U/μL	0.69 ± 0.02	0.68 ± 0.02	0.65 ± 0.01	0.70 ± 0.02	0.71 ± 0.05	0.68 ± 0.01
Intestine						
Endothelin-1, ng/g protein	22.33 ± 2.07	19.10 ± 0.76	21.56 ± 2.43	17.49 ± 2.04	22.13 ± 3.31	17.90 ± 0.50
Diamine oxidase, pg/g protein	124.41 ± 7.06	110.88 ± 2.76	122.71 ± 16.62	98.06 ± 10.45	121.44 ± 14.10	99.88 ± 0.13
Resistance value, kΩ	0.77 ± 0.02^a^	1.25 ± 0.05^b^	1.28 ± 0.05^b^	1.20 ± 0.04^b^	1.15 ± 0.02^b^	1.27 ± 0.04^b^

Values are presented as means ± SE (*n* = 3). ^a,b,c^ Means in the same row with different superscripts are significantly different (*P* < 0.05).

### 3.5 Antioxidant-related parameters

Dietary WBHPA supplementation resulted in significant reductions in the plasma ROS content and significant increases in intestinal GSH activity (*P* < 0.05; Table 7). However, the antioxidant-related parameters in the plasma and intestine were limitedly affected by dietary WBHPA supplementation. Compared with the control group, dietary 75 to 125 mg/kg astaxanthin supplementation markedly elevated the hepatic GSH-Px and GSH activities (*P* < 0.05). The increasing dietary astaxanthin inclusion resulted in initial elevations and subsequent reductions in the hepatic SOD and CAT (Fig. 1) activities, yielding the highest values in the WBHPA-75 and WBHPA-50 groups, respectively (*P* < 0.05). Conversely, the hepatic MDA (Fig. 1) and ROS contents firstly showed reductions and subsequently elevations with the increase of dietary WBHPA concentration, and the lowest values were observed in the WBHPA-50 group (*P* < 0.05).

**Table 7:** Antioxidant-related parameters in plasma, intestine, and liver of rainbow trout fed diets with various levels of astaxanthin from wall-broken *Haematococcus pluvialis* (WBHPA).

Item	Control	WBHPA-25	WBHPA-50	WBHPA-75	WBHPA-100	WBHPA-125
Plasma						
SOD, U/mL	89.23 ± 0.38	88.90 ± 0.22	89.73 ± 0.14	88.81 ± 0.46	88.99 ± 0.38	89.15 ± 0.33
CAT, U/mL	12.69 ± 1.96	14.00 ± 1.45	15.83 ± 1.99	15.97 ± 1.72	20.85 ± 3.02	19.76 ± 3.09
GSH-Px, μmol/L	126.16 ± 7.06	124.62 ± 17.31	128.46 ± 15.11	119.42 ± 13.27	143.46 ± 16.13	138.85 ± 6.67
GSH, μmol/L	23.87 ± 1.34	29.03 ± 2.07	28.17 ± 2.18	30.11 ± 1.14	33.33 ± 3.62	26.45 ± 1.62
TAC, U/mL	6.66 ± 0.49	7.32 ± 1.08	5.43 ± 0.62	5.84 ± 0.33	5.35 ± 0.70	5.75 ± 0.50
MDA, nmol/mL	15.57 ± 2.10	14.89 ± 2.10	13.92 ± 1.56	12.60 ± 1.92	7.96 ± 0.37	8.47 ± 1.46
ROS, U/μL	0.34 ± 0.01^c^	0.32 ± 0.01^bc^	0.33 ± 0.01^bc^	0.32 ± 0.02^bc^	0.20 ± 0.03^a^	0.25 ± 0.01^ab^
Intestine						
SOD, U/mg protein	25.99 ± 2.34	20.83 ± 0.64	22.51 ± 2.20	18.29 ± 1.93	25.41 ± 2.33	19.69 ± 0.53
CAT, U/mg protein	5.44 ± 0.43	5.94 ± 0.94	4.00 ± 0.13	4.58 ± 0.92	5.41 ± 0.85	4.52 ± 0.46
GSH-Px, μmol/g protein	30.56 ± 3.77	17.70 ± 0.97	19.32 ± 3.62	19.86 ± 3.46	22.57 ± 1.88	19.80 ± 1.19
GSH, μmol/g protein	2.20 ± 0.18^a^	10.25 ± 0.36^c^	5.17 ± 0.79^b^	2.57 ± 0.70^ab^	2.48 ± 0.05^ab^	2.21 ± 0.53^a^
TAC, U/mg protein	0.90 ± 0.12	1.08 ± 0.19	0.63 ± 0.09	0.66 ± 0.12	0.75 ± 0.07	0.79 ± 0.13
MDA, nmol/mg protein	25.51 ± 4.69	21.41 ± 0.92	17.77 ± 1.87	18.03 ± 3.67	14.74 ± 0.91	17.18 ± 1.21
ROS, U/mg protein	61.01 ± 6.51	48.54 ± 2.16	58.40 ± 4.78	51.51 ± 8.57	52.66 ± 6.29	50.90 ± 1.11
Liver						
SOD, U/mg protein	12.61 ± 0.53^a^	16.67 ± 0.88^abc^	17.01 ± 0.88^abc^	18.10 ± 0.14^c^	17.53 ± 1.30^bc^	13.37 ± 1.07^ab^
CAT, U/mg protein	20.85 ± 0.76^a^	25.35 ± 0.43^ab^	26.21 ± 0.50^b^	22.63 ± 1.70^ab^	22.20 ± 0.94^ab^	21.04 ± 1.02^a^
GSH-Px, μmol/g protein	4.92 ± 0.67^a^	5.30 ± 0.25^a^	7.88 ± 0.93^ab^	15.09 ± 1.48^bc^	16.10 ± 2.95^c^	16.53 ± 2.38^c^
GSH, μmol/g protein	6.34 ± 0.68^a^	8.32 ± 0.54^ab^	10.23 ± 0.81^b^	9.65 ± 0.09^b^	9.65 ± 0.45^b^	10.06 ± 0.67^b^
TAC, U/mg protein	0.85 ± 0.04	0.94 ± 0.14	0.85 ± 0.04	0.91 ± 0.04	0.92 ± 0.06	0.82 ± 0.03
MDA, nmol/mg protein	0.71 ± 0.02^b^	0.69 ± 0.04^ab^	0.53 ± 0.01^a^	0.64 ± 0.02^ab^	0.64 ± 0.04^ab^	0.75 ± 0.07^b^
ROS, U/mg protein	55.96 ± 2.09^b^	53.91 ± 2.06^ab^	45.23 ± 0.85^a^	48.91 ± 1.85^ab^	48.79 ± 1.23^ab^	46.41 ± 1.46^ab^

SOD = superoxide dismutase; CAT = catalase; GSH-Px = glutathione peroxidase; GSH = glutathione; TAC = total antioxidant capacity; MDA = malondialdehyde; ROS = reactive oxygen species.

Values are presented as means ± SE (*n* = 3). ^a,b,c^ Means in the same row with different superscripts are significantly different (*P* < 0.05).

### 3.6 Immune-related parameters

**Fig. 3:**
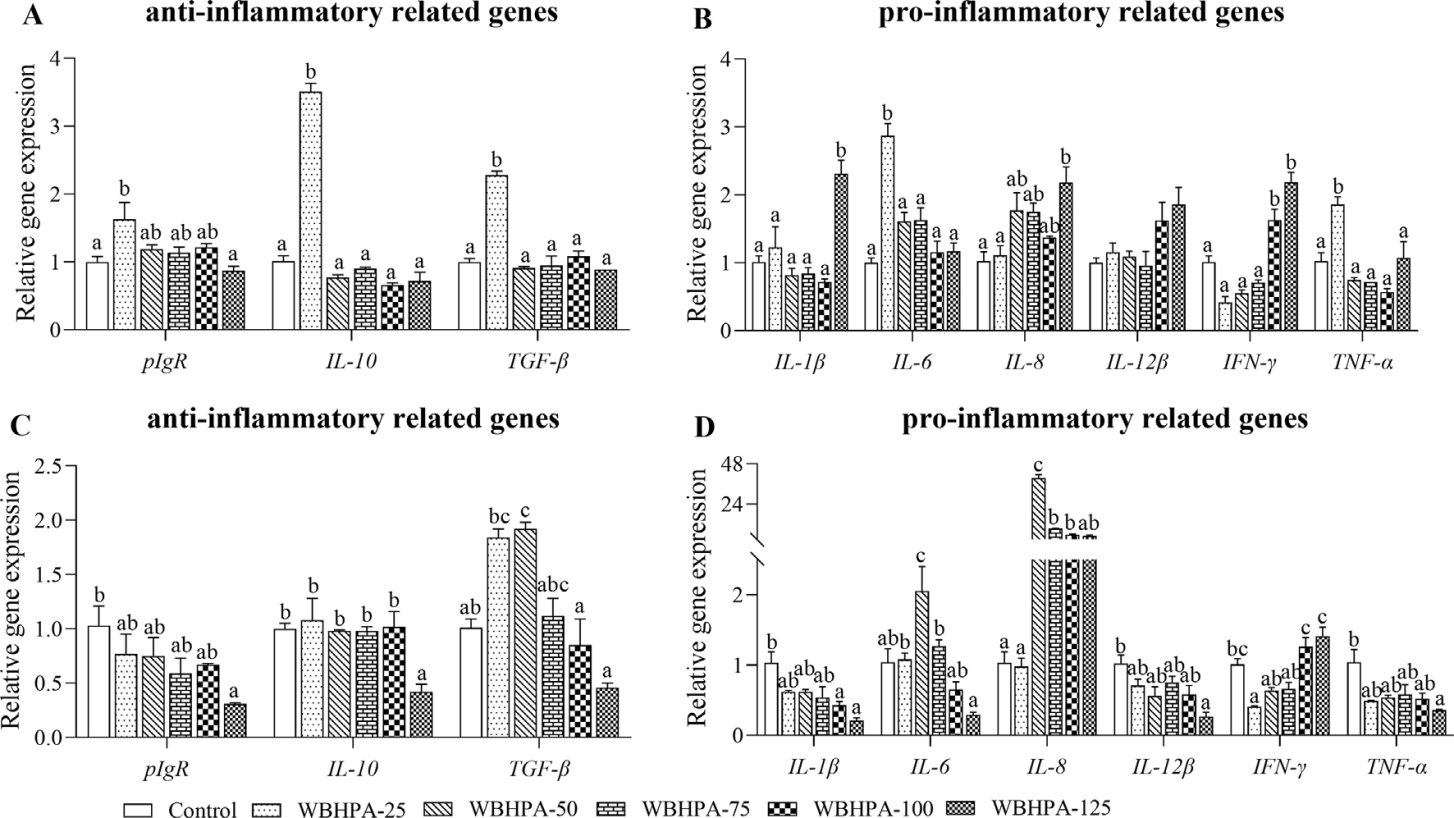
The relative expression (means ± SE) of (A) anti-inflammatory related genes in hindgut, (B) pro-inflammatory related genes in hindgut, (C) anti-inflammatory related genes in liver, and (D) pro-inflammatory related genes in liver of rainbow trout fed diets with various levels of astaxanthin from wall-broken *Haematococcus pluvialis* (WBHPA). *pIgR* = polymeric immunoglobulin receptor; *IL* = interleukin; *TGF-β1* = transforming growth factor-beta 1; *IFN-γ* = interferon-gamma; *TNF-α* = tumor necrosis factor-alpha. ^a,b,c^Bars with different superscript letters differ significantly (*P* < 0.05).

With the increase of dietary WBHPA concentration, the IL-10 and TGF-β contents in the hindgut first increased and then decreased (Table 8). Additionally, the IL-10 content in group WBHPA-50 and the TGF-β content in group WBHPA-75 were observed to be the highest among groups and were significantly higher than that in the control group (*P* < 0.05). Conversely, the IL-1β, IL-6, IL-8, IFN-γ, and TNF-α contents in the hindgut were firstly depressed and then raised with the increase of dietary WBHPA concentration, with the lowest values shown in the WBHPA-50 group, which were notably lower than the control group (*P* < 0.05). Compared with the control group, the transcription levels of hindgut polymeric immunoglobulin receptor (*pIgR*), *IL-10*, *TGF-β*, *IL-6*, and *TNF-α* in the WBHPA-25 group were significantly up-regulated (*P* < 0.05; Fig. 3). The relative levels of hindgut *IL-1β*, *IL-8*, and *IFN-γ* in the WBHPA-125 group were nearly two-fold those in the control group.

**Table 8:** Immune-related parameters in hindgut and liver of rainbow trout fed diets with various levels of astaxanthin from wall-broken *Haematococcus pluvialis* (WBHPA).

Item	Control	WBHPA-25	WBHPA-50	WBHPA-75	WBHPA-100	WBHPA-125
Hindgut						
IL-10, ng/g protein	26.57 ± 1.12^a^	32.12 ± 0.48^bc^	34.83 ± 0.69^c^	31.98 ± 1.09^bc^	29.25 ± 1.55^ab^	26.82 ± 1.48^ab^
TGF-β, ng/g protein	46.60 ± 1.06^a^	48.86 ± 0.45^ab^	50.48 ± 1.73^ab^	52.31 ± 0.40^b^	48.50 ± 0.88^ab^	46.94 ± 0.18^a^
IL-1β, ng/g protein	27.59 ± 0.59^b^	24.11 ± 1.04^ab^	21.39 ± 0.48^a^	23.78 ± 1.08^ab^	25.47 ± 0.89^ab^	26.37 ± 1.87^ab^
IL-6, ng/g protein	6.72 ± 0.38^c^	5.73 ± 0.12^ab^	5.15 ± 0.11^a^	6.14 ± 0.12^bc^	6.57 ± 0.15^bc^	6.49 ± 0.04^bc^
IL-8, ng/g protein	23.91 ± 0.27^b^	22.76 ± 0.85^ab^	19.70 ± 0.39^a^	20.72 ± 0.58^ab^	20.50 ± 1.05^ab^	20.78 ± 0.99^ab^
IFN-γ, ng/g protein	16.85 ± 0.70^c^	16.57 ± 0.40^bc^	13.90 ± 0.28^a^	14.33 ± 0.32^ab^	15.84 ± 0.76^abc^	16.40 ± 0.50^bc^
TNF-α, ng/g protein	16.18 ± 0.42^b^	15.25 ± 0.53^b^	12.94 ± 0.18^a^	14.22 ± 0.23^ab^	14.28 ± 0.76^ab^	15.01 ± 0.42^ab^
Liver						
IgM, mg/g protein	1.50 ± 0.04^a^	4.34 ± 0.41^bc^	3.84 ± 0.41^abc^	5.00 ± 0.53^c^	4.29 ± 0.93^bc^	2.41 ± 0.37^ab^
LZM, μg/g protein	17.27 ± 2.20	34.93 ± 5.11	31.94 ± 5.58	41.07 ± 8.43	22.52 ± 4.66	20.32 ± 4.85
IL-10, ng/g protein	21.87 ± 0.88	24.24 ± 0.67	20.99 ± 1.82	22.24 ± 1.47	23.00 ± 1.55	20.53 ± 0.91
TGF-β, μg/g protein	14.86 ± 0.75	16.93 ± 0.81	15.61 ± 1.41	16.05 ± 1.15	15.69 ± 0.90	15.40 ± 0.81
IL-1β, ng/g protein	16.62 ± 0.67	17.03 ± 0.59	15.02 ± 1.05	15.49 ± 0.71	16.33 ± 1.40	15.15 ± 1.05
IL-6, ng/g protein	3.58 ± 0.14	4.28 ± 0.20	3.87 ± 0.35	4.06 ± 0.24	4.03 ± 0.18	3.66 ± 0.21
IL-8, ng/g protein	18.47 ± 0.45	20.52 ± 0.28	18.21 ± 2.16	19.66 ± 0.78	20.68 ± 1.13	17.82 ± 0.76
IFN-γ, ng/g protein	82.47 ± 2.82	90.12 ± 2.55	90.93 ± 9.61	93.28 ± 5.13	93.67 ± 7.20	86.44 ± 3.55
TNF-α, ng/g protein	11.46 ± 0.32	13.16 ± 0.35	11.96 ± 1.00	12.58 ± 0.67	12.54 ± 0.99	11.03 ± 0.24

IL = interleukin; TGF-β = transforming growth factor-beta 1; IFN-γ = interferon-gamma; TNF-α = tumor necrosis factor-alpha; IgM = immunoglobulin M; LZM = lysozyme.

Values are presented as means ± SE (*n* = 3). ^a,b,c^ Means in the same row with different superscripts are significantly different (*P* < 0.05).

The hepatic IgM content and LZM activity were widely increased in a dose-dependent manner in response to dietary WBHPA inclusion (Table 8). Significant elevated IgM contents were detected in WBHPA-25, WBHPA-75, and WBHPA-100 groups versus the control group (*P* < 0.05). However, no significant difference was observed concerning the hepatic IL-10, TGF-β, IL-1β, IL-6, IL-8, IFN-γ, and TNF-α contents as well as the LZM activity (*P* > 0.05). The transcription levels of hepatic *pIgR*, *IL-10*, *IL-1β*, *IL-12β*, and *TNF-α* were generally down-regulated in response to increasing dietary WBHPA concentrations (Fig. 3). Meanwhile, the transcription of hepatic *TGF-β*, *IL-6*, and *IL-8* firstly increased and then decreased with the increase in dietary WBHPA concentration. The highest values of *TGF-β*, *IL-6*, and *IL-8* were observed in the WBHPA-50 group, which was significantly higher in comparison with the control group (*P* < 0.05). Conversely, the transcription of hepatic *IFN-γ* first decreased and then increased with the increase in dietary WBHPA concentration. The lowest value was determined in the WBHPA-25 group, which was significantly lower as compared to the control group (*P* < 0.05).

## 4 Discussion

In the present study, a 9-week feeding period with 25 to 100 mg/kg astaxanthin supplementation failed to cause significant differences in the rainbow trout (mean initial weight was 561 g), though the optimal growth performance was obtained in the WBHPA-50 group. Similar to this study, no significant differences were observed in some studies with coral trout *Plectropomus leopardus* (mean initial weight was 17.44 g; ≤2000 mg/kg *H. pluvialis*; Zhu et al., 2022), large yellow croaker (mean initial weight was 3 g; 250 to 500 mg/kg chemically synthesized astaxanthin [CSA, 10% purity, DSM]; Yi et al., 2018), and discus fish *Symphysodon* spp. (mean initial weight was 10.3 g; ≤200 mg/kg CSA [10% purity, BASF]; Song et al., 2017). However, several studies suggested that dietary inclusion of astaxanthin improved the growth performance in rainbow trout (mean initial weight was 27.75 g; 3300 mg/kg *H. pluvialis* or 1000 mg/kg CSA [10% purity, BASF]; Zhao et al., 2023), golden pompano *Trachinotus ovatus* (mean initial weight was 6.03 g; 500 to 1000 mg/kg CSA [10% purity, DSM]; Niu et al., 2020), large yellow croaker (mean initial weight was 5.57 g; 300 to 1000 mg/kg CSA [8% purity, Ningbo Honglong Biological Technology Co., Ltd.] or 2.8 to 11.2 g/kg *H. pluvialis*; Li et al., 2014), Asian seabass *Lates calcarifer* (mean initial weight was 28.07 g; 50 to 150 mg/kg astaxanthin from lyophilized *H. pluvialis*; Lim et al., 2019) and loach *Paramisgurnus dabryanus* (mean initial weight was 3 g; 50 to 200 mg/kg astaxanthin from *Chlorella vulgaris*; Chen et al., 2020). Dietary 30 mg/kg astaxanthin from *H. pluvialis* last for 4 month also significantly promoted the growth performance of rainbow trout with about 600 g initial weight (Long et al., 2023). Thus, the reason for insignificant advances in growth performance in the WBHPA-50 group may be also related to the fact that the 9-week experimental period was relatively short for rainbow trout with about 561 g initial weight. However, the present study showed that excess astaxanthin (125 mg/kg) supplementation significantly impaired the growth performance of rainbow trout, which was similar to the study on discus fish (300 to 400 mg/kg CSA; Song et al., 2017). The aforementioned disparity in the growth performance results may be linked to the basal diet, fish species, growth stage, and the culture environmental conditions.

To assess fish feed utilization, the activities of digestion and absorption enzymes and utilization of nutrients must be mentioned (Trenzado et al., 2018). Dietary 50 mg/kg astaxanthin supplementation increased intestinal creatine kinase activity, providing energy for the digestion and absorption of nutrients (Xu et al., 2022). Lipase is closely involved in intestinal lipid digestion (Zhang et al., 2020b), and intestinal AKP is highly implicated in the absorption of nutrients (Yuan et al., 2020). In this research, dietary 25 mg/kg astaxanthin supplementation contributed to the highest intestinal lipase and AKP activities. In contrast, trypsin and amylase activities displayed no noticeable change. Correspondingly, the WBHPA-25 group showed the best intestinal development (relative intestinal weight and intestinal density). Thus, in terms of the digestion and absorption capacity, dietary inclusion of 25 to 50 mg/kg astaxanthin was recommended. In addition, astaxanthin supplementation may promote intestinal development in rainbow trout by facilitating the digestion and absorption of nutrients, thereby contributing to better growth performance.

In addition to nutrient digestion and absorption, the intestine is also a critical immune organ. The intestinal mucosal barrier performs a significant role in systemic immune functions (Zhang et al., 2020a). As an intracellular enzyme, DAO extensively participates in catalyzing the oxidation of many diamines, which partly reflects the integrity and damage extent of the intestinal mechanical barrier (Dai et al., 2022). In this research, dietary WBHPA inclusion had no negative effect on the plasma and intestine DAO activities of rainbow trout. Furthermore, WBHPA supplementation in the diet demonstrated a markedly positive effect on the intestine resistance value and plasma ET-1 content, indicating the important role of astaxanthin in maintaining intestinal mucosal barrier integrity.

Besides the physical barrier function, intestinal epithelial cells also secrete cytokines that regulate immune responses and resist pathogen invasion (Zhao et al., 2021). The cytokines can mediate the inflammatory response, which is critical for the cellular immune response in fish (Whyte, 2007). It is well-known that anti-inflammatory cytokines (e.g., IL-10 and TGF-β) and pro-inflammatory cytokines (e.g., IL-1β, IL-6, IL-8, IL-12β, IFN-γ, and TNF-α) play crucial roles in the immune function of immune organs in fish (Pan et al., 2016). In this study, dietary WBHPA inclusion exhibited a markedly dose-dependent impact on the activities of intestinal anti-inflammatory and pro-inflammatory cytokines, and all the inflection points were present in the WBHPA-50 and WBHPA-75 groups. As an important member of the immunoglobulin superfamily, pIgR exerts a vital role in both innate and acquired immunity (Xia et al., 2022). In terms of genes related to anti-inflammatory function in the intestine, the transcription levels of *IL-10* and *TGF-β* were notably higher in the WBHPA-25 group than in the control group. Therein, the relative expression of hindgut *IL-10* in the WBHPA-25 group was nearly 3.5-fold that in the control group, indicating the powerful immune-promoting effect of astaxanthin. However, excessive dietary inclusion of astaxanthin (125 mg/kg) led to the down-regulation of anti-inflammatory cytokines and the up-regulation of pro-inflammatory cytokines, meaning damage to immune capacity. Thus, dietary inclusion of 25 to 75 mg/kg astaxanthin contributed to better intestinal immunity in fish.

Not only is the intestine the main immune organ of fish, but also the liver. As the metabolic center of nutrients, hepatic health is particularly important for fish health. IgM is the key to fish adaptive immune response (Rombout et al., 2014) and LZM is extensively distributed in multiple tissues and body fluids, which destroys and eliminates foreign bodies (Saurabh and Sahoo, 2008; Uribe et al., 2011). In the present study, with dietary WBHPA inclusion, the activities of both IgM and LZM were increased with their highest values in the WBHPA-75 group. Other studies also reported that the serum LZM activity of yellow croaker (Li et al., 2014) and common carp (Jagruthi et al., 2014) was also increased with dietary WBHPA inclusion. With dietary inclusion of 25 to 50 mg/kg astaxanthin, the relative expression of *TGF-β* was up-regulated. Meanwhile, the pro-inflammatory cytokines (e.g., *IL-1β*, *IL-12β*, *IFN-γ*, and *TNF-α*) were down-regulated with the inclusion of 25 to 75 mg/kg astaxanthin. These results also indicated that dietary 25 to 75 mg/kg astaxanthin inclusion can obtain better hepatic immunity in fish.

The biochemical parameters in the blood and tissues usually can reflect the antioxidant capacity of fish (Elia et al., 2019; Long et al., 2023). Overproduction of ROS leads to oxidative stress, further compromising cell function (Lushchak, 2016). As one of the important antioxidant enzymes, SOD, CAT, and GSH-Px can effectively protect cells from ROS-elicited damage (Messaoudi et al., 2009). SOD catalyzes the dismutation of superoxide radicals to hydrogen peroxide and oxygen, after which CAT catalyzes the breakdown of hydrogen peroxide to water and molecular oxygen, and GSH-Px decomposes peroxides with the peptide GSH as a cosubstrate (Tovar-Ramírez et al., 2010). TAC consists of the enzymatic (e.g. SOD, CAT, and GSH-Px) and non-enzymatic (e.g. ascorbate, urate, vitamin E, pyruvate, GSH, taurine, and hypo-taurine) antioxidants (Mahfouz et al., 2009; Yin et al., 2019). As the main component of lipid peroxides, MDA is usually considered a symbol of free radical destructive intensity (Rana et al., 2010). Astaxanthin is known for its powerful antioxidant properties. In order to comprehensively assess the impacts of dietary astaxanthin supplementation on the antioxidant status of rainbow trout, the above-mentioned antioxidant–relevant parameters in plasma, intestine, and liver were measured systematically in the present study. The hepatic SOD activity was remarkably up-regulated in the WBHPA-75 and WBHPA-100 groups. With the dietary inclusion of 50 mg/kg astaxanthin, the hepatic CAT and GSH levels were highest, while both MDA and ROS in the liver reached a minimum, indicating an improvement in antioxidant capacity. However, the lowest MDA contents in both plasma and intestine were observed in the WBHPA-100 group. In terms of the antioxidant response, the results in this research demonstrated that as the metabolic center of nutrients, the liver of rainbow trout was more sensitive to dietary astaxanthin supplementation than the plasma and intestine. Dietary supplementation with 30 mg/kg *H. pluvialis* astaxanthin lasting for 4 months has been suggested to improve the antioxidant capacity of commercial-sized rainbow trout (Long et al., 2023). Similarly, the results in the present study indicated that dietary inclusion of 25 to 125 mg/kg astaxanthin from WBHPA lasting for 9 weeks also exerted a better antioxidant capacity.

## 5 Conclusions

Dietary astaxanthin supplementation at the dosage range of 25 to 75 mg/kg has been shown to significantly enhance the integrity of the intestinal mucosal barrier and improve intestinal digestion and absorption capacity, thereby facilitating intestinal development. Moreover, it effectively strengthens the antioxidant capacity and immune response of rainbow trout. However, the excessive addition of astaxanthin (125 mg/kg) to the diet negatively impacts growth performance. Therefore, we recommend the inclusion of 25 to 75 mg/kg astaxanthin from WBHPA into the diet of rainbow trout with 561 g initial weight to achieve optimal antioxidant status, immune response, and intestinal health.

## Author contributions

**Xiaoxue Meng:** Software, Formal analysis, Writing – original draft. **Fumei Yang:** Investigation, Validation. **Lulu Zhu:** Formal analysis, Data curation. **Lingli Zhan:** Data curation, Methodology. **Toru Numasawa:** Funding acquisition, Supervision. **Junming Deng:** Writing – review & editing, Funding acquisition, Supervision, Conceptualization.

## Declaration of competing interest

We declare that we have no financial and personal relationships with other people or organizations that can inappropriately influence our work, and there is no professional or other personal interest of any nature or kind in any product, service and/or company that could be construed as influencing the content of this paper.
